# The radical scavenging activity of moracins: theoretical insights†

**DOI:** 10.1039/d0ra06555b

**Published:** 2020-10-06

**Authors:** Quan V. Vo, Nguyen Thi Hoa

**Affiliations:** Institute of Research and Development, Duy Tan University Danang 550000 Vietnam vovanquan2@duytan.edu.vn; The University of Danang – University of Technology and Education 48 Cao Thang Danang 550000 Vietnam vvquan@ute.udn.vn ngthoa@ute.udn.vn

## Abstract

Moracins are natural products that have been isolated from different plants such as *Artocarpus heterophyllus*, *Cassia fistula*, *Morus alba*, and *Morus mesozygia*. Studies showed that moracins may have various advantageous physiological effects such as anticancer, anti-inflammatory, anticholinesterase and particularly antioxidant activities. Most of these bioactivities have not been studied systematically. In this study, the radical scavenging of a typical moracin (moracin M, MM) against HO˙ and HOO˙ radicals was evaluated by thermodynamic and kinetic calculations in the gas phase as well as in water and pentyl ethanoate solvents. It was found that the overall rate constants for the HO˙ radical scavenging in the gas phase and the physiological environments are in the range of 10^11^ to 10^10^ M^−1^ s^−1^, respectively. For the HOO˙ + MM reaction the rate constants are 4.10 × 10^7^ and 3.80 × 10^4^ M^−1^ s^−1^ in the polar and lipid media, respectively. It is important to notice that the single electron transfer pathway of the anion state (MM–O6′^−^) dominated the HOO˙ radical scavenging in the aqueous solution, whereas in lipid medium the neutral MM exerted its activity by the formal hydrogen transfer mechanism. The HOO˙ radical scavenging of MM is comparable to that of Trolox in lipid medium, whereas it is 315.4 times more active in the polar environment.

## Abstract 2

The antiradical properties of moracin M in the gas-phase and in physiological environments were examined by thermodynamic and kinetic calculations.

## Graphical abstract

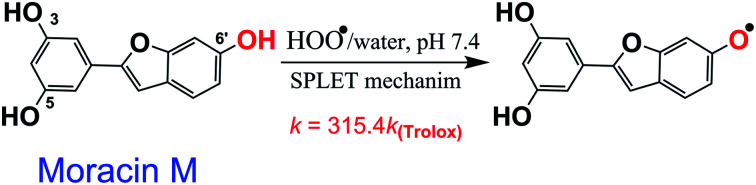

## 1. Introduction

The moracin family of natural product is based on a benzofuran heterocycle. There are about 24 natural moracins^1^ that have been isolated from a range of different plants such as *Artocarpus heterophyllus*,^2^*Cassia fistula*,^3^*Morus alba*,^4–7^ and *Morus mesozygia*.^8,9^ Studies showed that moracins can exert antiaromatase,^10^ anticancer,^11^ antidiabetes,^12^ anti-inflammatory,^13^ anticholinesterase,^14^ antifungal^15^ and antioxidant^9,16–18^ activities. The experimental data indicated that moracins have potent antioxidant activity.^1^ Moracins R, T and U showed good activity in 2,4-dinitrophenyl-1-picrylhydrazyl (DPPH) assays in methanol.^9^ Moracin C exerted high inhibitory activity in lipid peroxidase and free radical scavenging assays.^17,19^ Moracins M and N showed moderate free radical scavenging activity in inhibition of blue formazan formation and reduced the UV.^20^ In terms of theoretical studies, the antioxidant activity of moracin T was evaluated,^21^ however the research was limited to thermodynamic calculations. Kinetic analysis (*i.e.* calculating rate constants for the radical scavenging) is a more accurate way to predict activity and the effects of solvents, particularly the physiological environments, warranting further study.

**Fig. 1:**
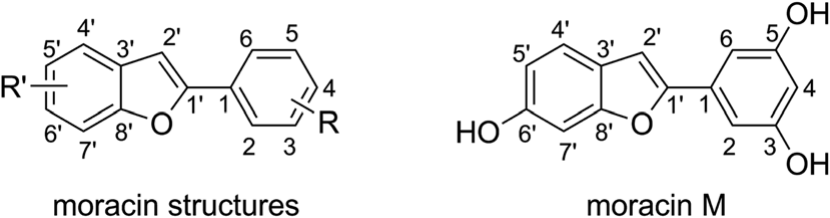
The generic structure of moracins and the structure of moracin M (MM).

The moracin structures are based on the benzofuran heterocycle (Fig. 1), in which the hydroxyl group mostly presents at C3, C5 and C6′ positions. Studies showed that the phenolic groups play a decisive role in the antioxidant activity of aromatic compounds.^22–27^ Moracin M (MM, Fig. 1)^28^ is a typical compound of the family since this compound contains HO groups in all of the typical positions but without any substituents. Considering that theoretical study on antioxidant activity of all of natural moracins is a difficult task due to the large structures and numerous compounds, in this study MM was used as a referenced compound for evaluating the antioxidant activity of moracins to save calculating time but still obtain reliable and accurate results.

Therefore, this study aims to investigate the radical scavenging activity of MM against HO˙ and HOO˙ radicals in the gas phase, as well as aqueous and lipid media using thermodynamic and kinetic calculations. The favored antioxidant mechanism of MM specific to each reactive oxygen species, chemical environments and moracin structures is also evaluated.

## 2. Computational methods

In this study, the quantum mechanics based test for overall free radical scavenging activity (QM-ORSA) protocol with the solvation model density (SMD) method (for water and pentyl ethanoate solvents) were used to performed the kinetic calculations.^22,29–34^ The rate constant (*k*) was calculated by using the conventional transition state theory (TST) (at 298.15 K, 1 M standard state) according to the eqn (1) (details method in Table S1, SI†):^35–40^1
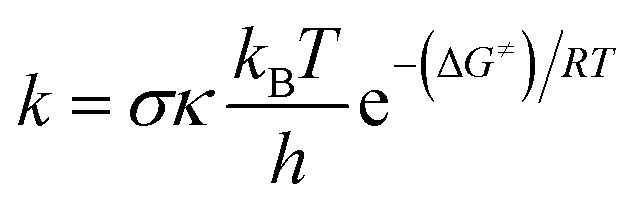
where: *σ* is the reaction symmetry number,^41,42^*κ* contains the tunneling corrections calculated using the Eckart barrier,^43^*k*_B_ is the Boltzmann constant, *h* is the Planck constant, Δ*G*^≠^ is the Gibbs free energy of activation.

All calculations were carried out using Gaussian 09 software^44^ at the M06-2X/6-311++G(d,p) level of theory.^24,45,46^

## 3. Results and discussions

### 3.1. The radical scavenging in the gas phase

#### 3.1.1. Thermodynamic study

Previous studies showed that the antioxidant activity could take place following either of three typical pathways including formal hydrogen transfer (FHT), sequential electron transfer proton transfer (SETPT), and sequential proton loss electron transfer (SPLET) mechanisms. From the thermodynamic point of view they can be characterized by the energetics of the respective first step of the mechanism, *i.e.* the bond dissociation enthalpy (BDE), ionization energy (IE) and proton affinity (PA) for FHT, SETPT and SPLET, respectively.^23,25^ Thus as an initial step, the thermochemical parameters of MM were calculated in the gas phase and are presented in the Table 1. The lowest calculated BDE and PA values were observed at the O6′–H bond at 83.3 and 336.6 kcal mol^−1^, respectively. The values of these parameters for O3(5)–H bonds were higher than that of the O6′–H bond by about 25 kcal mol^−1^ for BDEs and 4 kcal mol^−1^ for PAs. Thus it suggests that the antioxidant activity of MM according to FHT and SPLET mechanisms is dominated by the O6′–H bond. However, the antiradical activity of MM following the SETPT or SPLET would be difficult due to the high IE and PA values (IE = 170.9 kcal mol^−1^, PA = 336.6–340.2 kcal mol^−1^), compared with the BDE values. Thus these antioxidant mechanisms were ignored in further study and the antioxidant activity of MM was only modelled by H-abstraction at the O6′–H bond.

**Table 1:** The calculated BDEs, PAs and IEs (in kcal mol^−1^) in the gas phase of MM

Position	BDE	PA	IE
O3–H	108.3	340.2	170.9
O5–H	107.9	339.3	
O6′–H	83.3	336.6	

As shown in previous studies, there is an additional pathway to consider, the radical adduct formation (RAF) mechanism plays an important role in the radical scavenging of several phenolic compounds, particularly in the HO˙ antiradical activity.^31,47–49^ Thus, to gain further insights into the favored antioxidant pathways, the free energy (Δ*G*^o^) for the HO˙ and HOO˙ radicals scavenging of the MM in the gas phase following the FHT and RAF mechanisms were computed and are shown in Table 2. It was found that the HO˙ radical scavenging reactions are spontaneous (Δ*G*^o^ < 0) for all positions in MM, apart from the RAF at the C3′ position (Δ*G*^o^ = 3.5 kcal mol^−1^), whereas the HOO˙ radical scavenging is only spontaneous at the O6′–H bond (Δ*G*^o^ = −2.0 kcal mol^−1^) according to the FHT mechanism. Hence, the kinetic evaluation for the radical scavenging of MM against the HO˙ radical in vacuum was performed at all of positions (Δ*G*^o^ < 0), while that for the HOO˙ radical scavenging was only calculated for the H-abstraction of the O6′–H bond.

**Table 2:** The calculated Δ*G*^o^ values (in kcal mol^−1^) of the reactions of MM with HO˙ and HOO˙ following the FHT and RAF mechanisms in the gas phase

Mechanism	Position	Δ*G*^o^	
		OH	OOH
FHT	O6′	−33.3	−2.0
RAF	C1–OH	−2.0	19.0
	C2–OH	−15.2	7.1
	C3–OH	−8.3	12.6
	C4–OH	−14.0	7.7
	C5–OH	−8.0	12.1
	C6–OH	−17.1	6.9
	C1′–OH	−13.5	8.5
	C2′–OH	−17.7	5.4
	C3′–OH	3.5	21.5
	C4′–OH	−13.8	6.8
	C5′–OH	−8.3	11.5
	C6′–OH	−14.5	5.7
	C7′–OH	−10.6	7.8
	C8′–OH	−7.1	14.2

#### 3.1.2. Kinetic study

Kinetic study of the HO˙ and HOO˙ scavenging activity of MM in the gas phase was performed following the (QM-ORSA) protocol,^30,33,34^ and the kinetic parameters are presented in Table 3.

**Table 3:** Calculated activation energies Δ*G*^≠^ (kcal mol^−1^), tunneling corrections (*κ*) and *k*_Eck_ (M^−1^ s^−1^) at 298.15 K in the gas phase for the HO˙ and HOO˙ scavenging of the MM

Radical	Mechanism		Δ*G*^≠^	*κ*	*k*_Eck_	*Γ* b
HO˙	FHT	O6′	4.0	2.2	1.69 × 10^10^	15.5
	RAF	C1	13.3	1.4	1.57 × 10^3^	0.0
		C2	3.8	1.0	1.14 × 10^10^	10.5
		C3	9.8	1.3	4.94 × 10^5^	0.0
		C4	6.9	1.2	6.63 × 10^7^	0.1
		C5	9.4	1.3	1.08 × 10^6^	0.0
		C6	2.8	1.0	5.90 × 10^10^	54.4
		C1′	6.0	1.1	2.95 × 10^8^	0.3
		C2′	3.6	1.1	1.63 × 10^10^	15.0
		C4′	7.3	1.3	3.55 × 10^7^	0.0
		C5′	4.4	1.1	4.04 × 10^9^	3.7
		C6′	5.7	1.1	4.40 × 10^8^	0.4
		C7′	8.0	1.2	1.02 × 10^7^	0.0
		C8′	10.3	1.3	2.11 × 10^5^	0.0
	*k*_overall_ a				**1.08** × **10**^**11**^	
HOO˙	FHT	O6′	13.6	248.8	1.69 × 10^5^	100.0

a *k*_overall_ = ∑*k*_Eck_.

b *Γ* = *k*_Eck_ × 100/*k*_overall._

**Fig. 2:**
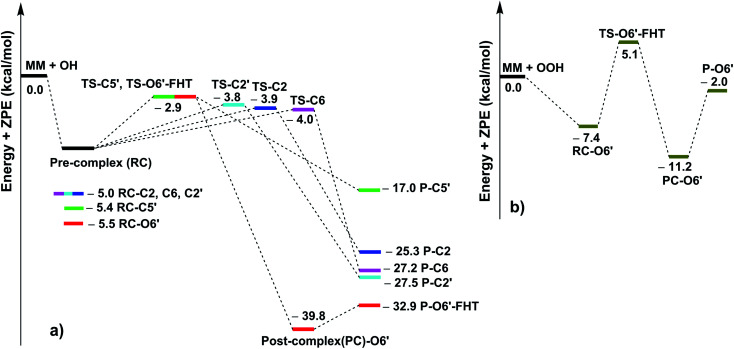
The PES of reaction according to FHT and RAF mechanisms between the MM and HO˙ (a) or HOO˙ (b) at the typical positions in the gas phase.

**Fig. 3:**
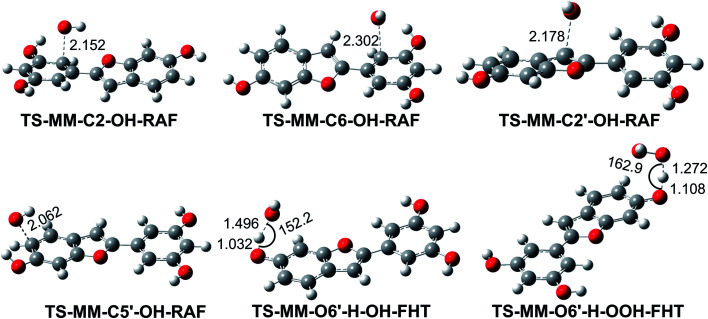
Optimized geometries of the typical transition states according to FHT and RAF mechanisms between the MM and HO˙/HOO˙ radicals in the gas phase.

As shown in Table 3, the HO˙ antiradical activity was dominated by the reactions at positions C2, C6, C2′ and C5′ for the RAF mechanism and the O6′–H bond for the FHT pathway as stated before. Thus the potential energy surfaces (PES) for these positions were also calculated and the results are shown in Fig. 2, whereas the optimized transition state (TS) structures and the density surfaces of the TSs and radicals are shown in Fig. 3 and S1,† respectively. Fig. 2 shows that the H-abstraction of O6′–H bond follows a typical radial reaction:^23,25^ reactant (R) → pre-complex (RC) → transition state (TS) → post-complex (PC) → product (P) where the calculated reaction barrier (energy + ZPE) was 4.3 kcal mol^−1^, whereas for the RAF mechanism at the C2, C6, C2′ and C5′ positions, the PC was not observed at the reaction line. The reaction barriers for RAF pathway were in the range of 1.0 to 2.5 kcal mol^−1^. The lowest reaction barrier was observed at the RAF of C6 position (1.0 kcal mol^−1^). This suggests that the addition of HO˙ radical at C6 plays a fundamental role in the hydroxyl radical scavenging of MM. In term of HOO˙ radicals, the reaction barrier for the H-abstraction of O6′–H bond was 12.5 kcal mol^−1^.

It was found that the overall rate constant (*k*_overall_) for the HO˙ radical scavenging in the gas phase was 1.08 × 10^11^ M^−1^ s^−1^, whereas that for the HOO˙ antiradical activity was 1.69 × 10^5^ M^−1^ s^−1^ (Table 3). The hydroxyl radical scavenging activity was defined by the RAF mechanism (*Γ* > 83%, at the C2, C6, C2′ and C5′ positions), in which the addition of HO˙ into C6 position contributed about 54% in the *k*_overall_. That is in good agreement with the obtained results at the PES analysis. The H-abstraction of O6′–H bond contributed 15.5% in the *k*_overall_ of the HO˙ radical scavenging, while that decided the HOO˙ antiradical activity.

### 3.2. The radical scavenging in the physiological environments

#### 3.2.1. Acid–base equilibria

To account for the effect of physiological environments, the radical scavenging of MM against HO˙ and HOO˙ radicals was modelled in water at pH = 7.4 for aqueous solution and in pentyl ethanoate for lipid medium. To determinate the state of MM in the aqueous solution at pH = 7.4, the acid–base equilibria of MM was calculated using the model reaction (2) and the eqn (3), given from literature.^24,30,50^

2 HA → A^−^ + H^+^

3 p*K*^calc^_a_ = *m*Δ*G*^o^_BA_ + *C*_0_

where Δ*G*^o^_BA_ was obtained from the reaction (2) following the eqn (4); *m* and *C*_0_ are fitted parameters directly obtained from ref. 50.4
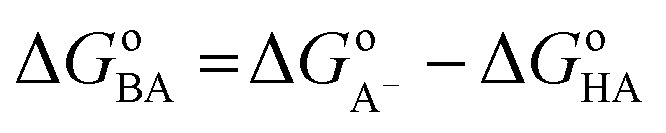


**Fig. 4:**
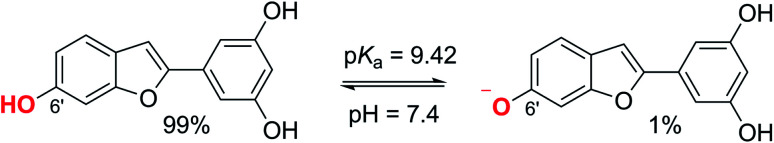
The deprotonation of MM.

As expected, the lowest PA value was calculated at O6′–H bond (Table 1). Thus this group was used to investigate the acid–base equilibria of MM. The calculated p*K*_a_ was 9.42. Consistently at physiological pH (7.40), MM exits both neutral state (MM, 99%) and monoanion state (MM–O6′^−^, 1%) (Fig. 4) and thus these states are used for further studies in the aqueous solution.

The reactivity of MM toward R˙ (R = HO˙ and HOO˙) radicals polar and nonpolar media were assessed by three typical antioxidant mechanisms: formal hydrogen transfer (FHT), single electron transfer (SET), and radical adduct formation (RAF). The processes can be described with the following reactions:^24,40^

5 MM + R˙ → MM^+^˙ + R^−^** **(SET-1)

6 MM–O6′^−^ + R˙ → MM–O6′˙ + R^−^** **(SET-2)

7 MM + R˙ → MM˙ + RH** **(FHT)

8 MM + R˙ → MM–R˙** **(RAF)

where R˙ = HO˙, HOO˙

#### 3.2.2. Kinetic study

Kinetics of the HO˙ and HOO˙ scavenging reactions in the physiological environments was investigated following the (QM-ORSA) protocol,^24,30^ and results are presented in Table 4. It was found that the *k*_overall_ for the HO˙ + MM reaction in water and pentyl ethanoate solvents were 2.73 × 10^10^ and 1.39 × 10^10^ M^−1^ s^−1^, respectively, whereas those for the HOO˙ + MM reaction were 4.10 × 10^7^ and 3.80 × 10^4^ M^−1^ s^−1^, respectively. The results showed that the HO˙ antiradical activity was defined by the RAF mechanism (*Γ* = 92.1% for the lipid medium and 65.5% for the aqueous solution). The SET pathway contributed about 29.4% of the overall rate constant in polar solvent, however this pathway had no contribution in the HO˙ radical scavenging of MM in the nonpolar environment. Compared to typical antioxidants such as melatonin,^51^ ramalin,^48^ indole-3-carbinol^23^ and Trolox,^30^ the hydroxyl radical scavenging of MM is in the range defined by these compounds in both polar and non-polar media.

**Table 4:** Gibbs free energies of activation (Δ*G*^≠^, kcal mol^−1^), rate constants (*k*_app_, *k*_f_, M^−1^ s^−1^) and branching ratios (*Γ*, %) at 298.15 K, in the MM oxidation by HO˙/HOO˙ radicals in the studied environments

Radical	Mechanism		Pentyl ethanoate			Water				
			Δ*G*^≠^	*k*_app_	*Γ*	Δ*G*^≠^	*k*_app_	*f*	*k*_f_ a	*Γ*
HO˙	SET-1		127.4	∼0	0.0	1.9	8.10 × 10^9^	0.99	8.02 × 10^9^	29.4
	SET-2					−13.1	8.30 × 10^9^	0.01	8.30 × 10^7^	0.3
	FHT	O6′	5.2	1.10 × 10^9^	7.9	4.8	1.30 × 10^9^	0.99	1.29 × 10^9^	4.7
	RAF	C2	3.0	2.50 × 10^9^	18.0	3.1	2.40 × 10^9^	0.99	2.38 × 10^9^	8.7
		C6	2.9	7.14 × 10^9^	51.3	3.0	6.85 × 10^9^	0.99	6.78 × 10^9^	24.9
		C2′	3.3	2.30 × 10^9^	16.5	2.4	7.80 × 10^9^	0.99	7.72 × 10^9^	28.3
		C5′	5.0	8.70 × 10^8^	6.3	4.9	1.00 × 10^9^	0.99	9.90 × 10^8^	3.6
	*k*_overall_			**1.39** × **10**^**10**^					**2.73** × **10**^**10**^	
HOO˙	SET-2				0.0	3.9	4.10 × 10^9^	0.01	4.10 × 10^7^	100
	HAT	O6′	14.7	3.80 × 10^4^	100	16.1	2.74 × 10^3^	0.99	2.72 × 10^3^	0.0
	*k*_overall_			**3.80** × **10**^**4**^					**4.10** × **10**^**7**^	

a *k*_f_ = *f* × *k*_app_.

It is important to notice that the single electron transfer pathway (SET-2) of the anion state (MM–O6′^−^) decided the HOO˙ radical scavenging in water at pH 7.4 despite of the fact that this state makes up only 1% of the total concentration under the given conditions. Compared with Trolox (*k*(HOO) = 1.30 × 10^5^ and 1.30 × 10^5^ M^−1^ s^−1^ in pentyl ethanoate and water, respectively)^24^ the HOO˙ radical scavenging activity of MM is slightly lower in lipid medium, however in the polar environment it is much higher (315.4 times) than that of Trolox. Thus MM is a promising radical scavenger especially in aqueous environment.

## 4. Conclusions

The antioxidant activity of MM was evaluated by thermodynamic and kinetic calculations in the gas phase as well as in physiological environments. It was found that the *k*_overall_ for the HO˙ radical scavenging in the gas phase was 1.08 × 10^11^ M^−1^ s^−1^, whereas that for the HOO˙ antiradical activity was 1.69 × 10^5^ M^−1^ s^−1^. In the polar and non-polar media, those for the HO˙ + MM reaction were about 10^10^ M^−1^ s^−1^, while for the HOO˙ + MM reaction, *k*_overall_ values were 4.10 × 10^7^ and 3.80 × 10^4^ M^−1^ s^−1^, respectively. It is important to notice that the single electron transfer pathway (SET-2) of the anion state (MM–O6′^−^) decided the HOO˙ radical scavenging in water at pH 7.4, while the HOO˙ radical scavenging of MM proceeded *via* the formal hydrogen transfer mechanism in the lipidic medium. Compared with typical antioxidants such as Trolox, the HOO˙ radical scavenging of MM is slightly lower in lipid medium but much higher (315.4 times) in water than that of Trolox. Thus MM is a promising radical scavenger in aqueous physiological environments.

## Conflicts of interest

There are no conflicts to declare.

## Supplementary Material

RA-010-D0RA06555B-s001
