# Supplementary material for: The radical scavenging activity of moracins: theoretical insights
Source: RSC Adv. 2020 Oct 6;10(60):36843–8. doi: 10.1039/d0ra06555b (PMC9057055; doi:10.1039/d0ra06555b)
Supplement: RA-010-D0RA06555B-s001 [file RA-010-D0RA06555B-s001.pdf]

Electronic Supplementary Material (ESI) for RSC Advances.
This journal is © The Royal Society of Chemistry 2020

Supporting Information (SI)

The Radical Scavenging Activity of Moracins: Theoretical Insights

Quan V. Vo[1,2*] and Nguyen Thi Hoa[2*]

[1]Institute of Research and Development, Duy Tan University, Danang 550000, Vietnam

[2]The University of Danang - University of Technology and Education, 48 Cao Thang, Danang 550000, Vietnam.

*Corresponding author: vovanquan2@duytan.edu.vn; vvquan@ute.udn.vn; ngthoa@ute.udn.vn

Table of Contents

Table S1. The method to calculate rate constant following the conventional transition state theory S2

Table S2: The Cartesian coordinates and energies of TS of the reaction between MM with $HO^•$/$HOO^•$ at the M06-2X/6-311++G(d,p) calculating method following the FHT and FHT mechanisms S3

Figure S1: The density surface of the typical TSs and radicals according to FHT and RAF.................................S20

Table S1. The method to calculate rate constant following the conventional transition state theory

The rate constant (k) was calculated by using the conventional transition state theory (TST) (at 298.15 K, 1M standard state) according to the equation (1):[1-5]

$$k = \sigma\kappa \frac{k_B T}{h} e^{-(\Delta G^{\neq})/RT} \qquad (1)$$

Where: σ is the reaction symmetry number ,[6,7]
κ contains the tunneling corrections calculated using the Eckart barrier,[8]
k_B is the Boltzmann constant,
h is the Planck constant,
$\Delta G^{\neq}$ is the Gibbs free energy of activation.

The Marcus Theory was used to estimate the reaction barriers of SET reactions.[9-12] The free energy of reaction $\Delta G^{\neq}$ for the SET pathway was computed following the equations (2,3).

$$\Delta G^{\neq}_{SET} = \frac{\lambda}{4}\left(1 + \frac{\Delta G^{0}_{SET}}{\lambda}\right)^2 \qquad (2)$$

$$\lambda \approx \Delta E_{SET} - \Delta G^{0}_{SET} \qquad (3)$$

where ΔG_{SET} is the Gibbs energy of reaction, ΔE_{SET} is the non-adiabatic energy difference between reactants and vertical products for SET.[13,14]

For rate constants that were close to the diffusion limit a correction was applied to yield realistic results[15]. The apparent rate constants (k_{app}) were calculated following the Collins–Kimball theory in the solvents at 298.15K;[16] the steady-state Smoluchowski rate constant (k_D) for an irreversible bimolecular diffusion–controlled reaction was calculated following the literature as corroding to equations (4,5).[15,17]

$$k_{app} = \frac{k_{TST} k_D}{k_{TST} + k_D} \qquad (4)$$

$$k_D = 4\pi R_{AB} D_{AB} N_A \qquad (5)$$

where R_{AB} is the reaction distance, N_A is the Avogadro constant, and $D_{AB} = D_A + D_B$ (D_{AB} is the mutual diffusion coefficient of the reactants A and B),[16,18] where D_A or D_B is estimated using the Stokes–Einstein formulation (6).[19,20]

$$D_{A\ or\ B} = \frac{k_B T}{6\pi\eta a_{A\ or\ B}} \qquad (6)$$

η is the viscosity of the solvents (i.e. $\eta(H_2O) = 8.91\times10^{-4}$ Pa s, η(pentyl ethanoate) = 8.62×10^{-4} Pa s) and a is the radius of the solute.

The kinetic study requires different considerations. Water (dielectric constants, ε = 78.35) and pentyl ethanoate (ε = 4.73) are the *de facto* standard solvents in the literature to mimic the polar and nonpolar environments in the human body.[15,21-23] Thus, these solvents were used to model the physiological environments. The solvent cage effects were included following the corrections proposed by Okuno,[24] adjusted with the free volume theory according to the Benson correction[15,25-27] to reduce over-penalizing entropy losses in solution. For the species that have multiple conformers, all of these were investigated and the conformer with the lowest electronic energy was included in the analysis.[22,23] The hindered internal rotation treatment was also applied to the single bonds to ensure that the obtained conformer has the lowest electronic energy.[23,28] All transition states were characterized by the existence of only one single imaginary frequency. Intrinsic coordinate calculations (IRCs) were performed to ensure that each transition state is connected correctly with the pre-complex and post-complex.

Table S2: The Cartesian coordinates and energies of TS of the reaction between MM with $HO^{\bullet}$/$HOO^{\bullet}$ at the M06-2X/6-311++G(d,p) calculating method following the FHT and FHT mechanisms

**Name**	**1-C1-OH-RAF (gas)**
Cartesian Coordinates	Frequency and Energy
O -0.84823900 0.75309300 0.20423000	Zero-point correction= 0.222324 (Hartree/Particle)
O -5.59591100 1.22969700 0.01747000	Thermal correction to Energy= 0.239053
O 4.53650100 -2.01511900 -0.21029400	Thermal correction to Enthalpy= 0.239998
O 3.79609600 2.59020100 -0.63563000	Thermal correction to Gibbs Free Energy= 0.177201
C -2.14217500 -1.04844800 -0.23703900	Sum of electronic and zero-point Energies= -915.805912
C -0.04118400 -0.33982500 0.03717000	Sum of electronic and thermal Energies= -915.789183
C -2.12457700 0.32359500 0.04344300	Sum of electronic and thermal Enthalpies= -915.788239
C 1.40156600 -0.10279300 0.16645700	Sum of electronic and thermal Free Energies= -915.851036
C -0.75791800 -1.45321800 -0.23913700	
C -3.37586900 -1.67030500 -0.43994000	
C -3.25719000 1.11376500 0.13552300	
C 1.90055400 1.19598200 -0.13959300	
C 2.28639600 -1.21354600 0.07144500	
C -4.46855400 0.46815500 -0.06998200	
C -4.52879000 -0.90714500 -0.35472800	
C 3.62837500 -1.00329100 -0.17446300	
C 3.24772800 1.37116000 -0.38626800	
C 4.12366900 0.28247500 -0.40221200	
H -0.36184300 -2.43636000 -0.43332700	
H -3.43838000 -2.72918200 -0.65898200	
H -3.21883100 2.17116400 0.35832600	
H 1.21083200 2.03152100 -0.13970000	
H 1.90675500 -2.20788500 0.27549200	
H -5.49741900 -1.37165500 -0.50827800	
H 5.17830800 0.43426500 -0.59118800	
H -6.37082000 0.68104600 -0.12728300	
H 4.10812700 -2.84708800 0.00834400	
H 3.11760100 3.26924600 -0.58584100	
O 1.33999100 0.00110700 2.12677900	
H 2.18598000 0.43372600 2.31793200	
**Name**	**1-C2-OH-RAF (gas)**
Cartesian Coordinates	Frequency and Energy
O 0.82116200 0.60397600 -0.04220600	Zero-point correction= 0.223662 (Hartree/Particle)
O 5.54293300 1.29923400 0.05072900	Thermal correction to Energy= 0.239930
O -3.81262100 2.30727500 -0.63293500	Thermal correction to Enthalpy= 0.240874
O -4.48524500 -2.23064300 0.34364300	Thermal correction to Gibbs Free Energy= 0.178965
C 2.22042000 -1.17327900 -0.08562200	Sum of electronic and zero-point Energies= -915.821560
C 0.06889400 -0.54794300 -0.09513100	Sum of electronic and thermal Energies= -915.805293
C 2.12412000 0.22345100 -0.03711700	Sum of electronic and thermal Enthalpies= -915.804348
C -1.37400300 -0.36439600 -0.11291200	Sum of electronic and thermal Free Energies= -915.866257
C 0.86227100 -1.64850000 -0.12493100	
C 3.49253500 -1.75227300 -0.09137600	
C 3.21084500 1.07867400 0.01083600	
C -2.22903300 -1.42280600 0.17019400	
C -1.90065900 0.90337900 -0.42765000	
C 4.46088100 0.47470400 0.00535300	
C 4.60074600 -0.92413900 -0.04524500	
C -3.30459700 1.07932600 -0.43293100	
C -3.61080800 -1.22900900 0.09425800	
C -4.15810100 0.01957400 -0.20048800	

H 0.52580600 -2.67049200 -0.19250800<br>H 3.61631500 -2.82748500 -0.13042600<br>H 3.11147200 2.15484400 0.04806900<br>H -1.83158100 -2.39145100 0.45354400<br>H -1.26936000 1.64756100 -0.89148000<br>H 5.59716800 -1.35372200 -0.04836600<br>H -5.22975700 0.16399600 -0.18942400<br>H 6.35094500 0.77970000 0.04886000<br>H -3.16855500 2.93343100 -0.26809700<br>H -4.01173800 -3.03435100 0.57578300<br>O -1.77294200 2.28508000 1.21702200<br>H -0.81808100 2.17802500 1.34058800	
**Name**	**1-C2-OH-RAF (water)**
Cartesian Coordinates	Frequency and Energy
O 0.83818500 0.66051400 -0.18298000<br>O 5.58604000 1.27794600 -0.07411100<br>O -3.85503000 2.37771800 -0.62832200<br>O -4.48465300 -2.20178600 0.15732900<br>C 2.21045100 -1.13375700 -0.02288500<br>C 0.06809400 -0.48706000 -0.13078800<br>C 2.13824900 0.26110300 -0.11664300<br>C -1.37562200 -0.29817900 -0.17681800<br>C 0.84639500 -1.59387200 -0.03403700<br>C 3.47286000 -1.73377100 0.05395700<br>C 3.23890300 1.10177800 -0.13645800<br>C -2.22620000 -1.38373500 0.01523500<br>C -1.90224800 0.98961900 -0.38178300<br>C 4.47561300 0.47485900 -0.05785300<br>C 4.59530800 -0.92392200 0.03592800<br>C -3.29549400 1.15639200 -0.42232600<br>C -3.60499400 -1.17922000 -0.02206400<br>C -4.15214800 0.08454300 -0.23662200<br>H 0.49760400 -2.61348500 0.02001700<br>H 3.57498900 -2.80970700 0.12721800<br>H 3.15193600 2.17827600 -0.20944300<br>H -1.83704600 -2.37972300 0.19397000<br>H -1.25685100 1.82645900 -0.61714800<br>H 5.58831400 -1.35620700 0.09493600<br>H -5.22582000 0.22332000 -0.25668500<br>H 6.38132700 0.73335100 -0.01653300<br>H -3.16800900 3.05049300 -0.72412500<br>H -4.00966200 -3.03104100 0.29827800<br>O -1.80836300 1.70039300 1.86665600<br>H -0.84122600 1.65132300 1.77988200	Zero-point correction= 0.221999 (Hartree/Particle)<br>Thermal correction to Energy= 0.238526<br>Thermal correction to Enthalpy= 0.239470<br>Thermal correction to Gibbs Free Energy= 0.176749<br>Sum of electronic and zero-point Energies= -915.860109<br>Sum of electronic and thermal Energies= -915.843582<br>Sum of electronic and thermal Enthalpies= -915.842638<br>Sum of electronic and thermal Free Energies= -915.905359
**Name**	**1-C2-OH-RAF (pentyl ethanoate)**
Cartesian Coordinates	Frequency and Energy
O 0.82246500 0.60696800 -0.08941000<br>O 5.54354500 1.29972700 0.02715000<br>O -3.82333000 2.30616800 -0.63842800<br>O -4.47769000 -2.23478600 0.32728800<br>C 2.22097100 -1.17085100 -0.06788200<br>C 0.07059500 -0.54655100 -0.11091500<br>C 2.12493400 0.22659300 -0.06250300<br>C -1.37360900 -0.36431100 -0.13385400	Zero-point correction= 0.222917 (Hartree/Particle)<br>Thermal correction to Energy= 0.239382<br>Thermal correction to Enthalpy= 0.240326<br>Thermal correction to Gibbs Free Energy= 0.177062<br>Sum of electronic and zero-point Energies= -915.848912<br>Sum of electronic and thermal Energies= -915.832448<br>Sum of electronic and thermal Enthalpies= -915.831504<br>Sum of electronic and thermal Free Energies= -915.894768

C	0.86308400	-1.64819100	-0.10059500	
C	3.49414700	-1.75116300	-0.04430000	
C	3.21128700	1.08416900	-0.03012400	
C	-2.22548000	-1.42855700	0.13989900	
C	-1.90213000	0.90572300	-0.43284500	
C	4.46095600	0.47843700	-0.00531200	
C	4.60247900	-0.92238900	-0.01312200	
C	-3.30151100	1.08208400	-0.42896200	
C	-3.60770100	-1.23111700	0.08336000	
C	-4.15624100	0.02216400	-0.19178100	
H	0.52621900	-2.67274800	-0.12778200	
H	3.61706500	-2.82755000	-0.04928300	
H	3.10784600	2.16154000	-0.02470200	
H	-1.82827000	-2.40389200	0.39972200	
H	-1.26748300	1.67431800	-0.85185000	
H	5.60056100	-1.34910200	0.00547900	
H	-5.22881600	0.16744600	-0.17641000	
H	6.35184300	0.77506900	0.04912400	
H	-3.16147600	2.95449700	-0.35706700	
H	-3.99935500	-3.04598900	0.53695200	
O	-1.78747400	2.27219000	1.32127300	
H	-0.82894300	2.14804100	1.40644800	
**Name**				**1-C3-OH-RAF (gas)**
Cartesian Coordinates				Frequency and Energy
O	-0.92865000	-0.66776400	-0.08408300	Zero-point correction= 0.222196 (Hartree/Particle)
O	-5.64697700	-1.38809200	0.00443500	Thermal correction to Energy= 0.238892
O	3.79526400	-2.25233500	-0.81783600	Thermal correction to Enthalpy= 0.239836
O	4.34862100	2.31494900	0.07699700	Thermal correction to Gibbs Free Energy= 0.176811
C	-2.33378800	1.10199300	-0.00393700	Sum of electronic and zero-point Energies= -915.811165
C	-0.18185000	0.48392300	-0.06947000	Sum of electronic and thermal Energies= -915.794469
C	-2.23211100	-0.29413200	-0.04438600	Sum of electronic and thermal Enthalpies= -915.793525
C	1.26736100	0.30895400	-0.11522900	Sum of electronic and thermal Free Energies= -915.856550
C	-0.97577800	1.58270700	-0.02448200	
C	-3.60730500	1.67495000	0.03909800	
C	-3.31546400	-1.15522900	-0.04161800	
C	2.11415800	1.41110700	0.04708900	
C	1.81246100	-0.94924700	-0.34095500	
C	-4.56753600	-0.55746000	0.00181300	
C	-4.71270400	0.84077800	0.04107100	
C	3.22765700	-1.12058500	-0.33147900	
C	3.50492300	1.25577600	-0.04505500	
C	4.06527200	0.01680100	-0.26223500	
H	-0.64710300	2.60930000	-0.01885200	
H	-3.73562700	2.74995000	0.06973800	
H	-3.21078900	-2.23106200	-0.07121600	
H	1.69632300	2.39429900	0.23699500	
H	1.17867700	-1.81357500	-0.48706200	
H	-5.71054600	1.26593800	0.07333500	
H	5.13673400	-0.10851700	-0.33495300	
H	-6.45614200	-0.87226500	0.04697300	
H	3.41822600	-2.99970100	-0.33833000	
H	3.84759300	3.12933100	0.16912700	
O	3.11252200	-1.82552800	1.50816100	
H	2.64463400	-1.17556600	2.05150200	

Name	1-C4-OH-RAF (gas)
Cartesian Coordinates	Frequency and Energy
O 1.04562400 0.74908600 -0.13498700<br>O 5.78069300 1.30425400 0.08756300<br>O -3.62079300 2.56190100 -0.35383500<br>O -4.31363000 -2.05885100 -0.38850400<br>C 2.38263700 -1.06629700 0.03737800<br>C 0.25566300 -0.37546800 -0.11458900<br>C 2.33165600 0.33168200 -0.04337300<br>C -1.18019800 -0.16056500 -0.19963300<br>C 1.01223200 -1.49949000 -0.00962800<br>C 3.63299300 -1.68254800 0.14063200<br>C 3.44547300 1.15304200 -0.03028200<br>C -2.04560700 -1.26173200 -0.26107800<br>C -1.68852000 1.14230200 -0.22516500<br>C 4.67282000 0.51279900 0.07259400<br>C 4.76656500 -0.88843300 0.15702000<br>C -3.05672000 1.33970400 -0.29959600<br>C -3.40825800 -1.04826600 -0.33038300<br>C -3.95069700 0.25079900 -0.28562200<br>H 0.64674100 -2.51232600 0.03696700<br>H 3.72070900 -2.75998500 0.20694300<br>H 3.38195100 2.23059300 -0.09367400<br>H -1.65170900 -2.27162500 -0.26146400<br>H -1.00948800 1.98552900 -0.19313100<br>H 5.74692100 -1.34674400 0.23642300<br>H -4.99437500 0.41221900 -0.51495500<br>H 6.56972300 0.76146300 0.16192700<br>H -2.94765600 3.24067200 -0.24994100<br>H -3.86124500 -2.90713100 -0.38478300<br>O -4.36705600 0.34296200 1.74269000<br>H -4.82050900 -0.51264500 1.78262800	Zero-point correction= 0.222958 (Hartree/Particle)<br>Thermal correction to Energy= 0.239628<br>Thermal correction to Enthalpy= 0.240572<br>Thermal correction to Gibbs Free Energy= 0.177395<br>Sum of electronic and zero-point Energies= -915.815726<br>Sum of electronic and thermal Energies= -915.799056<br>Sum of electronic and thermal Enthalpies= -915.798112<br>Sum of electronic and thermal Free Energies= -915.861289
**Name**	**1-C5-OH-RAF (gas)**
Cartesian Coordinates	Frequency and Energy
O 1.01887100 0.83445900 -0.10155400<br>O 5.78085200 1.16508500 0.01892300<br>O -3.57344600 2.85816700 0.16381600<br>O -4.44590100 -1.63592300 -0.83768800<br>C 2.27497800 -1.04469600 -0.01561300<br>C 0.18183300 -0.25496700 -0.09563200<br>C 2.28810600 0.35522300 -0.05455400<br>C -1.24838300 0.02874200 -0.13677400<br>C 0.88274700 -1.41421800 -0.03867800<br>C 3.49667300 -1.72006600 0.04025700<br>C 3.43865300 1.12398100 -0.04576900<br>C -2.15443700 -1.00144900 -0.38813500<br>C -1.71731000 1.32556000 0.06238300<br>C 4.63721600 0.42532900 0.00890100<br>C 4.66686700 -0.97979200 0.05176400<br>C -3.09906200 1.59553600 0.00546300<br>C -3.55465300 -0.74480500 -0.33385200<br>C -4.01011200 0.59125500 -0.21325700<br>H 0.46628900 -2.40773300 0.00092200<br>H 3.53513000 -2.80187300 0.07411700	Zero-point correction= 0.222436 (Hartree/Particle)<br>Thermal correction to Energy= 0.239107<br>Thermal correction to Enthalpy= 0.240051<br>Thermal correction to Gibbs Free Energy= 0.177401<br>Sum of electronic and zero-point Energies= -915.812271<br>Sum of electronic and thermal Energies= -915.795601<br>Sum of electronic and thermal Enthalpies= -915.794656<br>Sum of electronic and thermal Free Energies= -915.857307

H 3.42440600 2.20470500 -0.07918700	
H -1.82179900 -2.00925300 -0.59938800	
H -1.01152600 2.12788800 0.24623000	
H 5.62603100 -1.48535600 0.09495700	
H -5.07059300 0.79549100 -0.26281100	
H 6.54546000 0.58419800 0.04390500	
H -2.84343800 3.47704600 0.24984000	
H -4.32413700 -2.47045100 -0.36959400	
O -3.52732100 -1.56738400 1.44321700	
H -3.04896800 -0.96368300 2.02827700	
**Name**	**1-C6-OH-RAF (gas)**
Cartesian Coordinates	Frequency and Energy
O 0.97116200 1.04344400 -0.25233200	Zero-point correction= 0.221934 (Hartree/Particle)
O 5.80241200 1.10498900 -0.01344400	Thermal correction to Energy= 0.238919
O -3.80851200 2.90089800 0.45655300	Thermal correction to Enthalpy= 0.239863
O -4.44067300 -1.73105800 -0.42478100	Thermal correction to Gibbs Free Energy= 0.175870
C 2.14194400 -0.90826100 0.02409400	Sum of electronic and zero-point Energies= -915.821771
C 0.07208800 -0.03904300 -0.18821400	Sum of electronic and thermal Energies= -915.804786
C 2.24256600 0.49127800 -0.12707400	Sum of electronic and thermal Enthalpies= -915.803842
C -1.34935400 0.23955300 -0.21967800	Sum of electronic and thermal Free Energies= -915.867835
C 0.72522800 -1.21792600 -0.00855600	
C 3.31669900 -1.65131000 0.17239600	
C 3.44448600 1.17401700 -0.14391800	
C -2.22115500 -0.81971400 -0.52034100	
C -1.84923500 1.49707300 0.10278100	
C 4.60326500 0.41429400 0.00328400	
C 4.53478000 -0.98341400 0.16038900	
C -3.23701300 1.68365300 0.15908100	
C -3.60652100 -0.64671200 -0.33854300	
C -4.11061800 0.61028800 -0.02455800	
H 0.23312200 -2.16596700 0.14522500	
H 3.28119700 -2.72667600 0.29150200	
H 3.50495400 2.24462000 -0.26490600	
H -1.84376800 -1.69724800 -1.02480600	
H -1.15881700 2.30542700 0.31194800	
H 5.45436200 -1.54711800 0.27272800	
H -5.17295800 0.75753400 0.09750300	
H 6.56601100 0.48542100 0.08969000	
H -3.11660800 3.59720500 0.57985300	
H -3.87882500 -2.51538200 -0.13251300	
O -2.25211400 -2.76687100 0.70654800	
H -2.28983400 -2.21169000 1.54655800	
**Name**	**1-C6-OH-RAF (water)**
Cartesian Coordinates	Frequency and Energy
O 0.97116200 1.04344400 -0.25233200	Zero-point correction= 0.222248 (Hartree/Particle)
O 5.80241200 1.10498900 -0.01344400	Thermal correction to Energy= 0.237504
O -3.80851200 2.90089800 0.45655300	Thermal correction to Enthalpy= 0.238448
O -4.44067300 -1.73105800 -0.42478100	Thermal correction to Gibbs Free Energy= 0.179806
C 2.14194400 -0.90826100 0.02409400	Sum of electronic and zero-point Energies= -915.624171
C 0.07208800 -0.03904300 -0.18821400	Sum of electronic and thermal Energies= -915.608915
C 2.24256600 0.49127800 -0.12707400	Sum of electronic and thermal Enthalpies= -915.607971
C -1.34935400 0.23955300 -0.21967800	Sum of electronic and thermal Free Energies= -915.666613
C 0.72522800 -1.21792600 -0.00855600	
C 3.31669900 -1.65131000 0.17239600	

C 3.44448600 1.17401700 -0.14391800<br>C -2.22115500 -0.81971400 -0.52034100<br>C -1.84923500 1.49707300 0.10278100<br>C 4.60326500 0.41429400 0.00328400<br>C 4.53478000 -0.98341400 0.16038900<br>C -3.23701300 1.68365300 0.15908100<br>C -3.60652100 -0.64671200 -0.33854300<br>C -4.11061800 0.61028800 -0.02455800<br>H 0.23312200 -2.16596700 0.14522500<br>H 3.28119700 -2.72667600 0.29150200<br>H 3.50495400 2.24462000 -0.26490600<br>H -1.84376800 -1.69724800 -1.02480600<br>H -1.15881700 2.30542700 0.31194800<br>H 5.45436200 -1.54711800 0.27272800<br>H -5.17295800 0.75753400 0.09750300<br>H 6.56601100 0.48542100 0.08969000<br>H -3.11660800 3.59720500 0.57985300<br>H -3.87882500 -2.51538200 -0.13251300<br>O -1.73009788 -1.95525672 1.15707133<br>H -1.76781788 -1.40007572 1.99708133	
**Name**	**1-C1-OH-RAF (pentyl ethanoate)**
Cartesian Coordinates	Frequency and Energy
O 0.97116200 1.04344400 -0.25233200<br>O 5.80241200 1.10498900 -0.01344400<br>O -3.80851200 2.90089800 0.45655300<br>O -4.44067300 -1.73105800 -0.42478100<br>C 2.14194400 -0.90826100 0.02409400<br>C 0.07208800 -0.03904300 -0.18821400<br>C 2.24256600 0.49127800 -0.12707400<br>C -1.34935400 0.23955300 -0.21967800<br>C 0.72522800 -1.21792600 -0.00855600<br>C 3.31669900 -1.65131000 0.17239600<br>C 3.44448600 1.17401700 -0.14391800<br>C -2.22115500 -0.81971400 -0.52034100<br>C -1.84923500 1.49707300 0.10278100<br>C 4.60326500 0.41429400 0.00328400<br>C 4.53478000 -0.98341400 0.16038900<br>C -3.23701300 1.68365300 0.15908100<br>C -3.60652100 -0.64671200 -0.33854300<br>C -4.11061800 0.61028800 -0.02455800<br>H 0.23312200 -2.16596700 0.14522500<br>H 3.28119700 -2.72667600 0.29150200<br>H 3.50495400 2.24462000 -0.26490600<br>H -1.84376800 -1.69724800 -1.02480600<br>H -1.15881700 2.30542700 0.31194800<br>H 5.45436200 -1.54711800 0.27272800<br>H -5.17295800 0.75753400 0.09750300<br>H 6.56601100 0.48542100 0.08969000<br>H -3.11660800 3.59720500 0.57985300<br>H -3.87882500 -2.51538200 -0.13251300<br>O -1.74391387 -1.67118853 1.26471426<br>H -1.78163387 -1.11600753 2.10472426	Zero-point correction= 0.220939 (Hartree/Particle)<br>Thermal correction to Energy= 0.237619<br>Thermal correction to Enthalpy= 0.238563<br>Thermal correction to Gibbs Free Energy= 0.174115<br>Sum of electronic and zero-point Energies= -915.629751<br>Sum of electronic and thermal Energies= -915.613070<br>Sum of electronic and thermal Enthalpies= -915.612126<br>Sum of electronic and thermal Free Energies= -915.676574
**Name**	**1-C1’-OH-RAF (gas)**
Cartesian Coordinates	Frequency and Energy

O -0.72842100 -0.78063300 -0.23240700<br>O -5.46043200 -1.23805300 -0.58876900<br>O 4.02296700 -2.55138800 0.07713100<br>O 4.54699500 2.04564200 -0.65156100<br>C -2.06520000 1.02172000 0.10291800<br>C 0.05710800 0.27848300 0.11964100<br>C -2.01392200 -0.34918500 -0.19745100<br>C 1.50379100 0.09954000 -0.03387100<br>C -0.70771400 1.42227300 0.27756400<br>C -3.31558700 1.64479600 0.17830300<br>C -3.12671000 -1.13150400 -0.43408100<br>C 2.31897900 1.20289900 -0.28390400<br>C 2.04867500 -1.17547600 0.08984200<br>C -4.35415900 -0.48349900 -0.35796400<br>C -4.44877800 0.88837600 -0.05616200<br>C 3.42506900 -1.33711000 -0.03718400<br>C 3.69483400 1.01512500 -0.40222300<br>C 4.25571900 -0.24906500 -0.28176100<br>H -0.33163800 2.39776400 0.54086700<br>H -3.39994200 2.69771500 0.41685900<br>H -3.06583200 -2.18705900 -0.65961500<br>H 1.88802700 2.18943400 -0.41504400<br>H 1.40076600 -2.01524900 0.31216000<br>H -5.42942000 1.34974400 -0.00462600<br>H 5.32432900 -0.38601600 -0.38014700<br>H -6.25098700 -0.70070200 -0.49188300<br>H 3.35987200 -3.22405900 0.25494700<br>H 4.05376700 2.86623900 -0.73014800<br>O -0.10803400 -0.04500000 2.20993400<br>H 0.63382800 0.48339500 2.53999300	Zero-point correction= 0.222552 (Hartree/Particle)<br>Thermal correction to Energy= 0.239210<br>Thermal correction to Enthalpy= 0.240154<br>Thermal correction to Gibbs Free Energy= 0.177411<br>Sum of electronic and zero-point Energies= -915.817595<br>Sum of electronic and thermal Energies= -915.800936<br>Sum of electronic and thermal Enthalpies= -915.799992<br>Sum of electronic and thermal Free Energies= -915.862735
**Name**	**1-C2’-OH-RAF (gas)**
Cartesian Coordinates	Frequency and Energy
O 0.71124800 0.89373600 0.22532200<br>O 5.45929200 1.39517000 0.35095900<br>O -4.01068200 2.74520500 -0.05986000<br>O -4.61246900 -1.89971900 0.01702300<br>C 2.04821700 -0.80827000 -0.45073600<br>C -0.08378300 -0.14325200 -0.16920000<br>C 2.00518500 0.49481800 0.05311800<br>C -1.52112300 0.05750400 -0.10457500<br>C 0.66993000 -1.22762700 -0.56562700<br>C 3.29278400 -1.39018600 -0.68991100<br>C 3.12081400 1.25966500 0.33476600<br>C -2.36036700 -1.05757700 -0.06757400<br>C -2.04675000 1.35144300 -0.09342300<br>C 4.34844300 0.65707700 0.08258900<br>C 4.43424800 -0.65040900 -0.42443500<br>C -3.42842100 1.51644700 -0.06355500<br>C -3.73724400 -0.86174000 -0.03425100<br>C -4.28003100 0.41853300 -0.03796100<br>H 0.29237400 -2.08761800 -1.09062000<br>H 3.36986600 -2.40168500 -1.06836200<br>H 3.06270300 2.26589800 0.72631700<br>H -1.93102900 -2.05083700 0.00261800	Zero-point correction= 0.222849 (Hartree/Particle)<br>Thermal correction to Energy= 0.239588<br>Thermal correction to Enthalpy= 0.240532<br>Thermal correction to Gibbs Free Energy= 0.177742<br>Sum of electronic and zero-point Energies= -915.821460<br>Sum of electronic and thermal Energies= -915.804721<br>Sum of electronic and thermal Enthalpies= -915.803776<br>Sum of electronic and thermal Free Energies= -915.866567

H -1.38337100 2.20809600 -0.11961400	
H 5.41301000 -1.08305000 -0.60363200	
H -5.35240400 0.55871400 -0.00730900	
H 6.24816500 0.88668900 0.14564600	
H -3.33318000 3.42645600 -0.07136300	
H -4.12880600 -2.72923500 0.06062900	
O 0.24476700 -2.48300700 1.16345000	
H 0.63399200 -1.90306900 1.83519400	
**Name**	**1- C2’-OH-RAF (water)**
Cartesian Coordinates	Frequency and Energy
O 0.72006200 0.85926600 -0.04192300	Zero-point correction= 0.221589 (Hartree/Particle)
O 5.48072000 1.36235400 -0.07736500	Thermal correction to Energy= 0.238228
O -3.96716000 2.68354300 0.13200900	Thermal correction to Enthalpy= 0.239172
O -4.64500600 -1.93640500 -0.40276900	Thermal correction to Gibbs Free Energy= 0.176201
C 2.05436800 -0.96103100 -0.26402500	Sum of electronic and zero-point Energies= -915.861192
C -0.07640700 -0.25472700 -0.14705400	Sum of electronic and thermal Energies= -915.844552
C 2.01509600 0.42939400 -0.10837600	Sum of electronic and thermal Enthalpies= -915.843608
C -1.51810100 -0.03363900 -0.14693900	Sum of electronic and thermal Free Energies= -915.906580
C 0.68112400 -1.38764300 -0.27918000	
C 3.30176500 -1.59070900 -0.34680900	
C 3.13278100 1.24038500 -0.03867100	
C -2.37706900 -1.12868400 -0.27935200	
C -2.02547200 1.25929600 -0.00724800	
C 4.35579900 0.58588400 -0.13343800	
C 4.44323800 -0.81060400 -0.28198400	
C -3.40625700 1.44334200 -0.00319100	
C -3.74938900 -0.91048700 -0.27368400	
C -4.27741100 0.36989700 -0.13628400	
H 0.30571700 -2.39045100 -0.40956300	
H 3.37682600 -2.66506000 -0.46239700	
H 3.07180000 2.31468100 0.07810900	
H -1.99280400 -2.13707200 -0.38465700	
H -1.36687900 2.11271100 0.09872400	
H 5.42598200 -1.26482800 -0.34693200	
H -5.34924800 0.52602500 -0.13272100	
H 6.26576000 0.80817300 -0.17182400	
H -3.27507200 3.34958300 0.23033200	
H -4.17413500 -2.77443200 -0.49399800	
O 0.07862600 -1.23311400 2.19519000	
H 1.04972500 -1.26852000 2.23121300	
**Name**	**1- C2’-OH-RAF (pentyl ethanoate)**
Cartesian Coordinates	Frequency and Energy
O 0.71107400 0.88804800 0.18858300	Zero-point correction= 0.222425 (Hartree/Particle)
O 5.46017900 1.39312000 0.26756100	Thermal correction to Energy= 0.239173
O -4.00100000 2.73605400 0.00109500	Thermal correction to Enthalpy= 0.240117
O -4.62062900 -1.90659300 -0.10044100	Thermal correction to Gibbs Free Energy= 0.177101
C 2.04583500 -0.83754400 -0.42696000	Sum of electronic and zero-point Energies= -915.848953
C -0.08434200 -0.16255400 -0.15905400	Sum of electronic and thermal Energies= -915.832205
C 2.00456500 0.48421300 0.02724100	Sum of electronic and thermal Enthalpies= -915.831261
C -1.52206500 0.04279100 -0.10602800	Sum of electronic and thermal Free Energies= -915.894277
C 0.66807900 -1.26195400 -0.51274200	
C 3.29127900 -1.42783900 -0.65220800	
C 3.12052600 1.26183300 0.27231300	
C -2.36644800 -1.06994900 -0.11942500	

C -2.04350500 1.33786700 -0.05898400	
C 4.34766500 0.64931900 0.03642600	
C 4.43297900 -0.67762800 -0.42127400	
C -3.42590900 1.50662100 -0.03804200	
C -3.74282300 -0.87111500 -0.09728300	
C -4.28204300 0.41150100 -0.06018500	
H 0.28831000 -2.15874700 -0.97191700	
H 3.36885500 -2.45145700 -0.99831600	
H 3.05905100 2.28346500 0.62418200	
H -1.95153600 -2.07132800 -0.10347000	
H -1.38309700 2.19742400 -0.05139600	
H 5.41233600 -1.11362500 -0.58974900	
H -5.35534500 0.55436400 -0.03899100	
H 6.24846700 0.87361100 0.07211800	
H -3.31786300 3.41600700 0.02393900	
H -4.13953300 -2.74223000 -0.09571200	
O 0.22521600 -2.27480500 1.40956600	
H 0.90887200 -1.78744900 1.89563600	
**Name**	**1-C3'-OH-RAF (gas)**
Cartesian Coordinates	Frequency and Energy
O 0.57487700 0.83774500 0.20184600	Zero-point correction= 0.223092 (Hartree/Particle)
O 5.26085300 1.53418300 -0.18576500	Thermal correction to Energy= 0.239541
O -4.10538900 2.63713100 0.09216100	Thermal correction to Enthalpy= 0.240485
O -4.76401400 -1.98986500 -0.20308000	Thermal correction to Gibbs Free Energy= 0.178000
C 1.94525800 -0.95539100 -0.10925700	Sum of electronic and zero-point Energies= -915.807599
C -0.19750000 -0.27856900 -0.08134700	Sum of electronic and thermal Energies= -915.791150
C 1.86340000 0.45888500 0.09339800	Sum of electronic and thermal Enthalpies= -915.790206
C -1.64343600 -0.07115100 -0.06201800	Sum of electronic and thermal Free Energies= -915.852691
C 0.56412600 -1.35863500 -0.33673700	
C 3.19196400 -1.46596200 -0.57870300	
C 2.95070900 1.29874000 0.11271400	
C -2.50064100 -1.17093800 -0.13413500	
C -2.15988000 1.22081600 0.01852300	
C 4.18000900 0.71938800 -0.20594500	
C 4.29172700 -0.64234000 -0.57424000	
C -3.54176600 1.40245400 0.01717700	
C -3.87547200 -0.96278900 -0.13732000	
C -4.40664600 0.32023700 -0.06305000	
H 0.22026700 -2.35206500 -0.57035400	
H 3.27446500 -2.50620400 -0.86583400	
H 2.87232100 2.35850400 0.31174100	
H -2.10102400 -2.17820500 -0.16762900	
H -1.48980800 2.06989600 0.07923400	
H 5.26225400 -1.02719600 -0.86978200	
H -5.47789100 0.47063600 -0.05973500	
H 6.04832100 1.04714700 -0.44439600	
H -3.41760600 3.30428700 0.16388600	
H -4.29381700 -2.82726000 -0.23132000	
O 2.05695800 -1.54439600 1.71262200	
H 2.94512000 -1.24637900 1.95755200	
**Name**	**1-C4'-OH-RAF (gas)**
Cartesian Coordinates	Frequency and Energy
O 0.45905900 0.94549100 0.04362600	Zero-point correction= 0.222765 (Hartree/Particle)
O 5.16858700 1.73447600 -0.00753400	Thermal correction to Energy= 0.239483

O -4.29578200 2.53301600 0.26816200<br>O -4.76201900 -2.10891600 -0.12289400<br>C 1.87464600 -0.76636900 -0.36719000<br>C -0.27550100 -0.19362600 -0.16021300<br>C 1.76569600 0.59816900 -0.07732900<br>C -1.72521100 -0.05470400 -0.07650700<br>C 0.53434800 -1.25935700 -0.41250400<br>C 3.16400100 -1.33575400 -0.49146100<br>C 2.84245400 1.45382500 0.05233100<br>C -2.53622800 -1.19053100 -0.13991600<br>C -2.29503200 1.21050400 0.06241100<br>C 4.10993200 0.88620900 -0.12782000<br>C 4.26894500 -0.46365700 -0.42481200<br>C -3.68153200 1.32852800 0.13385900<br>C -3.91746100 -1.04532100 -0.06882500<br>C -4.50052000 0.20973300 0.06744100<br>H 0.22090500 -2.27224700 -0.60373900<br>H 3.28942500 -2.32945200 -0.89595400<br>H 2.73450200 2.50605000 0.27802200<br>H -2.09675100 -2.17726600 -0.23316700<br>H -1.66136800 2.08785200 0.11174200<br>H 5.26572200 -0.86895900 -0.55914700<br>H -5.57596000 0.31129100 0.12459100<br>H 5.99036200 1.24759800 -0.11240300<br>H -3.63747700 3.23165300 0.31186000<br>H -4.25914900 -2.92323300 -0.20737900<br>O 3.27757600 -2.29886700 1.32758300<br>H 3.17919600 -1.52078700 1.89724100	Thermal correction to Enthalpy= 0.240427<br>Thermal correction to Gibbs Free Energy= 0.176984<br>Sum of electronic and zero-point Energies= -915.814834<br>Sum of electronic and thermal Energies= -915.798117<br>Sum of electronic and thermal Enthalpies= -915.797173<br>Sum of electronic and thermal Free Energies= -915.860615
**Name**	**1-C5'-OH-RAF (gas)**
Cartesian Coordinates	Frequency and Energy
O 0.35344600 0.83765900 -0.08273500<br>O 5.08292700 1.48898400 -0.18113400<br>O -4.34929700 2.54757400 0.16011700<br>O -4.94354400 -2.09533600 0.11248200<br>C 1.73711200 -0.93881600 -0.31231800<br>C -0.40910100 -0.31058800 -0.13884100<br>C 1.64602400 0.46367900 -0.18940400<br>C -1.85395000 -0.12679200 -0.04192700<br>C 0.37378100 -1.40712800 -0.28054900<br>C 2.98863800 -1.51749000 -0.46283700<br>C 2.73377800 1.32248500 -0.15615200<br>C -2.69521400 -1.24167800 -0.00856700<br>C -2.38787600 1.15974200 0.01543100<br>C 3.97737100 0.72564600 -0.26967700<br>C 4.12383400 -0.68950000 -0.42812900<br>C -3.76976600 1.32044300 0.10221400<br>C -4.07050400 -1.05428300 0.07627700<br>C -4.61828400 0.22271800 0.13261000<br>H 0.03597900 -2.42738500 -0.35834700<br>H 3.10650400 -2.58322700 -0.61189100<br>H 2.63644800 2.38962500 -0.01287600<br>H -2.28444500 -2.24448700 -0.04296500<br>H -1.73159500 2.02122800 -0.00943100<br>H 5.06528200 -1.06469500 -0.80466400	Zero-point correction= 0.223629 (Hartree/Particle)<br>Thermal correction to Energy= 0.239925<br>Thermal correction to Enthalpy= 0.240869<br>Thermal correction to Gibbs Free Energy= 0.178362<br>Sum of electronic and zero-point Energies= -915.819962<br>Sum of electronic and thermal Energies= -915.803666<br>Sum of electronic and thermal Enthalpies= -915.802722<br>Sum of electronic and thermal Free Energies= -915.865229

H -5.68951000 0.35713600 0.19998400<br>H 5.75146700 0.94807800 0.26635700<br>H -3.67350900 3.22997300 0.12705100<br>H -4.46522800 -2.92710200 0.06349400<br>O 5.13254400 -0.83976300 1.36356800<br>H 4.38493700 -0.78271700 1.97610600	
**Name**	**1-C5’-OH-RAF (water)**
Cartesian Coordinates	Frequency and Energy
O 0.39640000 0.84876700 -0.13983200<br>O 5.12168800 1.46176900 -0.37389800<br>O -4.31806900 2.56198600 0.27409500<br>O -4.90902300 -2.09520500 -0.00921500<br>C 1.75867800 -0.95529300 -0.29762900<br>C -0.38038800 -0.30220100 -0.15538400<br>C 1.68927500 0.45174000 -0.22749500<br>C -1.82443100 -0.10882400 -0.06273100<br>C 0.39129600 -1.40995200 -0.25003300<br>C 3.00382500 -1.55695700 -0.39849100<br>C 2.78340200 1.29726500 -0.25018800<br>C -2.66489400 -1.22609200 -0.08974600<br>C -2.35559200 1.17672400 0.05650300<br>C 4.02020100 0.67361600 -0.34384000<br>C 4.14454400 -0.74294000 -0.37837600<br>C -3.73732100 1.32922000 0.15162400<br>C -4.03799500 -1.04007600 0.00759400<br>C -4.58908000 0.23226700 0.12801600<br>H 0.04331800 -2.43045600 -0.27893300<br>H 3.10922600 -2.63246200 -0.46710000<br>H 2.69318800 2.37447000 -0.20169000<br>H -2.26462900 -2.22911500 -0.18473700<br>H -1.71434900 2.04939000 0.07956300<br>H 5.12240500 -1.16701600 -0.57056000<br>H -5.66136800 0.36443000 0.20474400<br>H 5.91922400 0.91574800 -0.39363000<br>H -3.63725800 3.24647100 0.29779800<br>H -4.41737300 -2.92378800 -0.07809100<br>O 4.68538400 -0.77172400 1.77465200<br>H 3.78745300 -0.52340100 2.04727400	Zero-point correction= 0.222123 (Hartree/Particle)<br>Thermal correction to Energy= 0.238612<br>Thermal correction to Enthalpy= 0.239556<br>Thermal correction to Gibbs Free Energy= 0.177185<br>Sum of electronic and zero-point Energies= -915.857636<br>Sum of electronic and thermal Energies= -915.841148<br>Sum of electronic and thermal Enthalpies= -915.840203<br>Sum of electronic and thermal Free Energies= -915.902575
**Name**	**1-C5’-OH-RAF (pentyl ethanoate)**
Cartesian Coordinates	Frequency and Energy
O 0.36250600 0.83498000 -0.14475200<br>O 5.08842200 1.46578600 -0.32120700<br>O -4.33489100 2.55285800 0.24756300<br>O -4.94197500 -2.08541700 0.01142800<br>C 1.73755900 -0.95867100 -0.25307000<br>C -0.40456700 -0.31262100 -0.12588500<br>C 1.65284600 0.44952100 -0.22513300<br>C -1.84988200 -0.12112700 -0.04027700<br>C 0.37286600 -1.41998600 -0.18995300<br>C 2.98682400 -1.55198300 -0.35879400<br>C 2.74304300 1.30529600 -0.25672100<br>C -2.69395700 -1.23510500 -0.05878100<br>C -2.37901000 1.16576100 0.06090100<br>C 3.98356000 0.69468300 -0.33067900	Zero-point correction= 0.223184 (Hartree/Particle)<br>Thermal correction to Energy= 0.239525<br>Thermal correction to Enthalpy= 0.240469<br>Thermal correction to Gibbs Free Energy= 0.178239<br>Sum of electronic and zero-point Energies= -915.846647<br>Sum of electronic and thermal Energies= -915.830306<br>Sum of electronic and thermal Enthalpies= -915.829362<br>Sum of electronic and thermal Free Energies= -915.891592

C 4.12457100 -0.72755400 -0.37738700<br>C -3.76139800 1.32693600 0.14562100<br>C -4.06907100 -1.04563200 0.02602800<br>C -4.61421500 0.23092400 0.12915300<br>H 0.03090100 -2.44264900 -0.18897500<br>H 3.10011200 -2.62663300 -0.42995700<br>H 2.64580200 2.38134300 -0.19878400<br>H -2.28841300 -2.23742100 -0.14124900<br>H -1.72601000 2.03026400 0.07386200<br>H 5.07146900 -1.13701700 -0.70363600<br>H -5.68652800 0.36669100 0.19415400<br>H 5.79752800 0.94703400 0.08632400<br>H -3.65263800 3.23417000 0.24947500<br>H -4.46032800 -2.91702600 -0.06438400<br>O 5.06445700 -0.74715800 1.48446500<br>H 4.28494500 -0.56978200 2.03306100	
**Name**	**1-C6'-OH-RAF (gas)**
Cartesian Coordinates	Frequency and Energy
O 0.43975500 0.70425400 -0.15265900<br>O 5.24175800 0.99041100 -0.60394500<br>O -4.18411100 2.64948100 -0.12209500<br>O -4.98367200 -1.94612100 0.26094200<br>C 1.72963700 -1.15709800 -0.19228400<br>C -0.37282500 -0.39232100 -0.10067500<br>C 1.72030700 0.24629300 -0.20899700<br>C -1.80836600 -0.13766000 -0.04101600<br>C 0.35565700 -1.54542700 -0.11952300<br>C 2.96567300 -1.83267100 -0.26359200<br>C 2.85317000 1.02317500 -0.29773200<br>C -2.69839600 -1.20708100 0.08585600<br>C -2.28457800 1.17101900 -0.11187500<br>C 4.08863700 0.33512600 -0.29230300<br>C 4.11691200 -1.09066400 -0.32937300<br>C -3.65822700 1.39917300 -0.05700900<br>C -4.06466000 -0.95277800 0.13755800<br>C -4.55476400 0.34658800 0.06681400<br>H -0.03797800 -2.54833700 -0.09478200<br>H 3.00351800 -2.91477000 -0.27199200<br>H 2.82621000 2.10379700 -0.34009600<br>H -2.33282700 -2.22578900 0.14860800<br>H -1.58954400 1.99619500 -0.20989700<br>H 5.09247200 -1.55582900 -0.38257800<br>H -5.61943000 0.53348800 0.10899600<br>H 5.45108100 1.56118800 0.14675200<br>H -3.47931600 3.29715300 -0.20743300<br>H -4.54313900 -2.79893000 0.30225600<br>O 4.40022500 0.70788700 1.73531300<br>H 3.58824700 0.47048900 2.20463600	Zero-point correction= 0.222210 (Hartree/Particle)<br>Thermal correction to Energy= 0.238997<br>Thermal correction to Enthalpy= 0.239941<br>Thermal correction to Gibbs Free Energy= 0.176205<br>Sum of electronic and zero-point Energies= -915.817086<br>Sum of electronic and thermal Energies= -915.800299<br>Sum of electronic and thermal Enthalpies= -915.799355<br>Sum of electronic and thermal Free Energies= -915.863091
**Name**	**1-C7'-OH-RAF (gas)**
Cartesian Coordinates	Frequency and Energy
O 0.53508200 0.56869300 -0.32075500<br>O 5.28619400 0.87286700 -0.60503600<br>O -4.02741400 2.66195100 -0.09294500<br>O -4.96188000 -1.92537300 0.04323000	Zero-point correction= 0.223239 (Hartree/Particle)<br>Thermal correction to Energy= 0.239799<br>Thermal correction to Enthalpy= 0.240743<br>Thermal correction to Gibbs Free Energy= 0.177867

C 1.79795500 -1.24810900 0.09148300<br>C -0.30065100 -0.48109200 -0.03004500<br>C 1.79495700 0.10050100 -0.24747700<br>C -1.73192000 -0.19165400 -0.03988400<br>C 0.41152000 -1.60764500 0.22941600<br>C 3.03044500 -1.91073100 0.20812700<br>C 2.95048800 0.87594300 -0.42328400<br>C -2.65359800 -1.24022100 -0.00302000<br>C -2.17087700 1.13070600 -0.07538300<br>C 4.16015100 0.15369500 -0.36577700<br>C 4.19864900 -1.20301500 -0.02210100<br>C -3.53916200 1.39412500 -0.06489400<br>C -4.01397400 -0.95036000 0.00919100<br>C -4.46792200 0.36320100 -0.02227100<br>H 0.00737000 -2.56777200 0.50568000<br>H 3.07450500 -2.96008900 0.47216800<br>H 2.91853600 1.85739500 -0.87349100<br>H -2.31533600 -2.27053000 0.00108300<br>H -1.45040900 1.93969300 -0.09895300<br>H 5.15907700 -1.70133600 0.05561800<br>H -5.52814800 0.57759000 -0.01469700<br>H 6.06270600 0.31504900 -0.50308100<br>H -3.29980300 3.28965800 -0.10492800<br>H -4.54112300 -2.78867000 0.06671300<br>O 3.06059900 1.71769900 1.43340900<br>H 3.97561100 2.03024900 1.36617900	Sum of electronic and zero-point Energies= -915.814110<br>Sum of electronic and thermal Energies= -915.797549<br>Sum of electronic and thermal Enthalpies= -915.796605<br>Sum of electronic and thermal Free Energies= -915.859482
**Name**	**1-C8’-OH-RAF (gas)**
Cartesian Coordinates	Frequency and Energy
O 0.59768000 0.70415800 -0.26365100<br>O 5.34049000 1.08893400 -0.72600600<br>O -4.05172400 2.58703300 0.00473400<br>O -4.78008300 -2.03310200 -0.22640500<br>C 1.91503500 -1.10179100 0.14023600<br>C -0.19172700 -0.38413200 -0.03091600<br>C 1.88121000 0.31075400 -0.05235200<br>C -1.63181300 -0.16034900 -0.06816000<br>C 0.55074400 -1.50399400 0.20738800<br>C 3.15860600 -1.74223300 0.17663600<br>C 3.01438600 1.04530800 -0.45809500<br>C -2.50418900 -1.24927200 -0.13915300<br>C -2.12744800 1.14178100 -0.02433500<br>C 4.21558600 0.37992100 -0.42468500<br>C 4.29117300 -0.99782700 -0.08961000<br>C -3.50555100 1.34420900 -0.04281400<br>C -3.87554700 -1.02001400 -0.15578500<br>C -4.38616200 0.27249300 -0.10868800<br>H 0.16585200 -2.48643100 0.42586600<br>H 3.23394600 -2.80440900 0.37251500<br>H 2.95435300 2.09630100 -0.70339700<br>H -2.11889800 -2.26115500 -0.19741600<br>H -1.44132300 1.97769500 0.04151000<br>H 5.26560400 -1.47589200 -0.07135300<br>H -5.45489000 0.43964100 -0.12295000<br>H 6.10124300 0.50342600 -0.75218700	Zero-point correction= 0.222692 (Hartree/Particle)<br>Thermal correction to Energy= 0.239265<br>Thermal correction to Enthalpy= 0.240209<br>Thermal correction to Gibbs Free Energy= 0.177791<br>Sum of electronic and zero-point Energies= -915.810846<br>Sum of electronic and thermal Energies= -915.794273<br>Sum of electronic and thermal Enthalpies= -915.793329<br>Sum of electronic and thermal Free Energies= -915.855746

H -3.35503800 3.24604200 0.06819600<br>H -4.32383900 -2.87837900 -0.24298400<br>O 1.82637000 0.79643600 1.88574600<br>H 2.68531700 0.48638500 2.20885700	
**Name**	**1-O6'-OH-FHT (gas)**
Cartesian Coordinates	Frequency and Energy
O -0.48787000 0.51869300 -0.22081400<br>O -5.28479600 0.55313900 -0.33307000<br>O 4.00625900 2.74156200 -0.21934200<br>O 5.08458000 -1.78842000 0.25166700<br>C -1.67863300 -1.37977700 0.02575000<br>C 0.38270700 -0.51343500 -0.02933800<br>C -1.74022700 -0.00193000 -0.18226700<br>C 1.80206100 -0.17683300 -0.01747200<br>C -0.28863900 -1.69334100 0.12284400<br>C -2.88644300 -2.12129200 0.04267800<br>C -2.91507000 0.70902800 -0.37805600<br>C 2.75619700 -1.18922100 0.11252100<br>C 2.19831800 1.15520900 -0.13240400<br>C -4.11644300 -0.04883100 -0.28989500<br>C -4.07631300 -1.46209900 -0.11812500<br>C 3.55671800 1.46494700 -0.11321800<br>C 4.10586900 -0.85358900 0.12886000<br>C 4.51646600 0.47009300 0.01706100<br>H 0.15392400 -2.66285900 0.28320500<br>H -2.86779600 -3.19435600 0.19071600<br>H -2.92094100 1.74042900 -0.69751300<br>H 2.45346700 -2.22694300 0.19592500<br>H 1.45382600 1.93581700 -0.23259900<br>H -5.02457300 -1.98238300 -0.08146300<br>H 5.56883100 0.72021500 0.03051900<br>H -5.09022500 1.46065200 0.11745900<br>H 3.26293100 3.34523900 -0.30153600<br>H 4.69622300 -2.66469400 0.31757400<br>O -4.22732400 2.38143700 0.92137500<br>H -4.11186100 2.12401300 1.84554500	Zero-point correction= 0.220414 (Hartree/Particle)<br>Thermal correction to Energy= 0.236800<br>Thermal correction to Enthalpy= 0.237744<br>Thermal correction to Gibbs Free Energy= 0.174426<br>Sum of electronic and zero-point Energies= -915.819953<br>Sum of electronic and thermal Energies= -915.803567<br>Sum of electronic and thermal Enthalpies= -915.802623<br>Sum of electronic and thermal Free Energies= -915.865940
**Name**	**1-O6'-OH-FHT (water)**
Cartesian Coordinates	Frequency and Energy
O -0.48787000 0.51869300 -0.22081400<br>O -5.28479600 0.55313900 -0.33307000<br>O 4.00625900 2.74156200 -0.21934200<br>O 5.08458000 -1.78842000 0.25166700<br>C -1.67863300 -1.37977700 0.02575000<br>C 0.38270700 -0.51343500 -0.02933800<br>C -1.74022700 -0.00193000 -0.18226700<br>C 1.80206100 -0.17683300 -0.01747200<br>C -0.28863900 -1.69334100 0.12284400<br>C -2.88644300 -2.12129200 0.04267800<br>C -2.91507000 0.70902800 -0.37805600<br>C 2.75619700 -1.18922100 0.11252100<br>C 2.19831800 1.15520900 -0.13240400<br>C -4.11644300 -0.04883100 -0.28989500<br>C -4.07631300 -1.46209900 -0.11812500<br>C 3.55671800 1.46494700 -0.11321800	Zero-point correction= 0.219152 (Hartree/Particle)<br>Thermal correction to Energy= 0.233658<br>Thermal correction to Enthalpy= 0.234602<br>Thermal correction to Gibbs Free Energy= 0.177115<br>Sum of electronic and zero-point Energies= -915.860742<br>Sum of electronic and thermal Energies= -915.846236<br>Sum of electronic and thermal Enthalpies= -915.845292<br>Sum of electronic and thermal Free Energies= -915.902778

C	4.10586900	-0.85358900	0.12886000	
C	4.51646600	0.47009300	0.01706100	
H	0.15392400	-2.66285900	0.28320500	
H	-2.86779600	-3.19435600	0.19071600	
H	-2.92094100	1.74042900	-0.69751300	
H	2.45346700	-2.22694300	0.19592500	
H	1.45382600	1.93581700	-0.23259900	
H	-5.02457300	-1.98238300	-0.08146300	
H	5.56883100	0.72021500	0.03051900	
H	-5.09022500	1.46065200	0.11745900	
H	3.26293100	3.34523900	-0.30153600	
H	4.69622300	-2.66469400	0.31757400	
O	-4.24076884	2.35819177	0.90542582	
H	-4.12530584	2.10076777	1.82959582	
**Name**				**1-O6’-OH-FHT (pentyl ethanoate)**
Cartesian Coordinates				Frequency and Energy
O	-0.49461300	0.54173200	-0.22215700	Zero-point correction= 0.220737 (Hartree/Particle)
O	-5.28900100	0.60647600	-0.33911400	Thermal correction to Energy= 0.236933
O	4.02912300	2.71778900	-0.26188500	Thermal correction to Enthalpy= 0.237878
O	5.05057300	-1.81744000	0.29832900	Thermal correction to Gibbs Free Energy= 0.175601
C	-1.69336200	-1.35304300	-0.00052500	Sum of electronic and zero-point Energies= -915.846163
C	0.37061100	-0.49737500	-0.03826400	Sum of electronic and thermal Energies= -915.829967
C	-1.74756900	0.02616800	-0.19302600	Sum of electronic and thermal Enthalpies= -915.829022
C	1.79334600	-0.17087000	-0.02049500	Sum of electronic and thermal Free Energies= -915.891299
C	-0.30532200	-1.67626100	0.09817000	
C	-2.90309600	-2.09222400	-0.00111800	
C	-2.92174200	0.74380300	-0.38390300	
C	2.73305800	-1.19384800	0.13524700	
C	2.20469800	1.15557500	-0.15685800	
C	-4.12391600	-0.01698300	-0.31130400	
C	-4.09309100	-1.42766700	-0.15955900	
C	3.56697000	1.44870200	-0.13511800	
C	4.08666200	-0.87286300	0.15235600	
C	4.51459100	0.44444000	0.01846300	
H	0.13301700	-2.65067900	0.24553000	
H	-2.88707900	-3.16706900	0.13550400	
H	-2.92144200	1.76599400	-0.73208400	
H	2.41833400	-2.22650700	0.23983400	
H	1.47732900	1.94966700	-0.27684300	
H	-5.03975200	-1.95241800	-0.13031700	
H	5.57087400	0.68216500	0.03369900	
H	-5.07520300	1.47044900	0.16460900	
H	3.28894500	3.32831800	-0.35803300	
H	4.64644400	-2.68867400	0.38239600	
O	-4.10999800	2.26705200	1.06673400	
H	-3.99115700	1.85855700	1.93605400	
**Name**				**1-O6’-OOH-FHT (gas)**
Cartesian Coordinates				Frequency and Energy
O	0.00387300	0.74540100	-0.25215800	Zero-point correction= 0.223270 (Hartree/Particle)
O	-4.72438400	1.11749400	-0.79597900	Thermal correction to Energy= 0.240866
O	4.63410000	2.64424800	0.10012200	Thermal correction to Enthalpy= 0.241810
O	5.36943000	-1.96593900	0.42553400	Thermal correction to Gibbs Free Energy= 0.174705
C	-1.28990300	-1.10533400	-0.44866000	Sum of electronic and zero-point Energies= -990.965254
C	0.80629300	-0.35996200	-0.20017100	Sum of electronic and thermal Energies= -990.947658

C	-1.27368300	0.30253900	-0.40017800	Sum of electronic and thermal Enthalpies= -990.946714
C	2.23524400	-0.12064800	-0.03948000	Sum of electronic and thermal Free Energies= -991.013820
C	0.07433600	-1.50575400	-0.31778100	
C	-2.52174100	-1.76845100	-0.60008100	
C	-2.39713000	1.08912900	-0.48080100	
C	3.10617500	-1.20185100	0.11996100	
C	2.72527500	1.18518900	-0.04723200	
C	-3.62496500	0.41488500	-0.65143900	
C	-3.66459900	-1.01350600	-0.69940700	
C	4.09421600	1.39856100	0.10135300	
C	4.46813700	-0.96211900	0.26546300	
C	4.97185100	0.33419500	0.25756600	
H	0.46160300	-2.51142800	-0.31731800	
H	-2.56674800	-2.84978400	-0.63402500	
H	-2.37099400	2.16980300	-0.44207500	
H	2.72822200	-2.21778700	0.13714300	
H	2.04463200	2.01880600	-0.17120600	
H	-4.63677500	-1.47791200	-0.81000900	
H	6.03309300	0.50971400	0.37272900	
H	-5.46978700	0.83235000	-0.02688400	
H	3.94237800	3.30139500	-0.01494000	
H	4.92184100	-2.81605600	0.41544900	
H	-4.71774600	0.31036500	2.38316500	
O	-6.02204900	0.53599800	1.07989000	
O	-5.08931500	-0.25104100	1.68675300	
**Name**				**1-O6'-OOH-FHT (water)**
Cartesian Coordinates				Frequency and Energy
O	0.00387300	0.74540100	-0.25215800	Zero-point correction= 0.222136 (Hartree/Particle)
O	-4.72438400	1.11749400	-0.79597900	Thermal correction to Energy= 0.237598
O	4.63410000	2.64424800	0.10012200	Thermal correction to Enthalpy= 0.238542
O	5.36943000	-1.96593900	0.42553400	Thermal correction to Gibbs Free Energy= 0.176818
C	-1.28990300	-1.10533400	-0.44866000	Sum of electronic and zero-point Energies= -991.005752
C	0.80629300	-0.35996200	-0.20017100	Sum of electronic and thermal Energies= -990.990290
C	-1.27368300	0.30253900	-0.40017800	Sum of electronic and thermal Enthalpies= -990.989346
C	2.23524400	-0.12064800	-0.03948000	Sum of electronic and thermal Free Energies= -991.051071
C	0.07433600	-1.50575400	-0.31778100	
C	-2.52174100	-1.76845100	-0.60008100	
C	-2.39713000	1.08912900	-0.48080100	
C	3.10617500	-1.20185100	0.11996100	
C	2.72527500	1.18518900	-0.04723200	
C	-3.62496500	0.41488500	-0.65143900	
C	-3.66459900	-1.01350600	-0.69940700	
C	4.09421600	1.39856100	0.10135300	
C	4.46813700	-0.96211900	0.26546300	
C	4.97185100	0.33419500	0.25756600	
H	0.46160300	-2.51142800	-0.31731800	
H	-2.56674800	-2.84978400	-0.63402500	
H	-2.37099400	2.16980300	-0.44207500	
H	2.72822200	-2.21778700	0.13714300	
H	2.04463200	2.01880600	-0.17120600	
H	-4.63677500	-1.47791200	-0.81000900	
H	6.03309300	0.50971400	0.37272900	
H	-5.46978700	0.83235000	-0.02688400	
H	3.94237800	3.30139500	-0.01494000	

H	4.92184100	-2.81605600	0.41544900	
H	-4.72137139	0.30874043	2.38840577	
O	-6.02567439	0.53437343	1.08513077	
O	-5.09294039	-0.25266557	1.69199377	
**Name**				**1-O6'-OOH-FHT (pentyl ethanoate)**
Cartesian Coordinates				Frequency and Energy
O	-0.04363200	0.69263900	-0.30948100	Zero-point correction= 0.223130 (Hartree/Particle)
O	-4.78026000	0.93356600	-0.90617200	Thermal correction to Energy= 0.240638
O	4.56259900	2.68168500	-0.05079200	Thermal correction to Enthalpy= 0.241582
O	5.35635000	-1.89460200	0.53537600	Thermal correction to Gibbs Free Energy= 0.175129
C	-1.29650900	-1.19207900	-0.40902600	Sum of electronic and zero-point Energies= -990.996663
C	0.77987600	-0.39155300	-0.19108000	Sum of electronic and thermal Energies= -990.979154
C	-1.30942600	0.21585500	-0.44058000	Sum of electronic and thermal Enthalpies= -990.978210
C	2.20418500	-0.11842000	-0.03914200	Sum of electronic and thermal Free Energies= -991.044663
C	0.07319300	-1.55835800	-0.24692800	
C	-2.51406700	-1.88927300	-0.53359600	
C	-2.44767400	0.97427700	-0.57890900	
C	3.08687400	-1.18008700	0.18080700	
C	2.67473400	1.19328600	-0.11747300	
C	-3.65782000	0.26379200	-0.71348400	
C	-3.67078800	-1.16329500	-0.68201200	
C	4.04081900	1.43164100	0.02090800	
C	4.44502700	-0.91307000	0.31758500	
C	4.93211900	0.38836800	0.23874400	
H	0.48290800	-2.55424400	-0.18619900	
H	-2.53510900	-2.97202900	-0.51047800	
H	-2.43850700	2.05669900	-0.60467200	
H	2.72177200	-2.19907900	0.25005100	
H	1.98959700	2.01538600	-0.28771600	
H	-4.63084500	-1.65662300	-0.78037200	
H	5.99167000	0.58459000	0.34646800	
H	-5.41873800	0.84114100	-0.02699600	
H	3.85892900	3.32266400	-0.20500100	
H	4.91528800	-2.75109800	0.57285100	
H	-4.32853700	0.79667900	2.27916500	
O	-5.84834200	0.75410500	1.20204200	
O	-4.82817600	0.10128300	1.81877800	

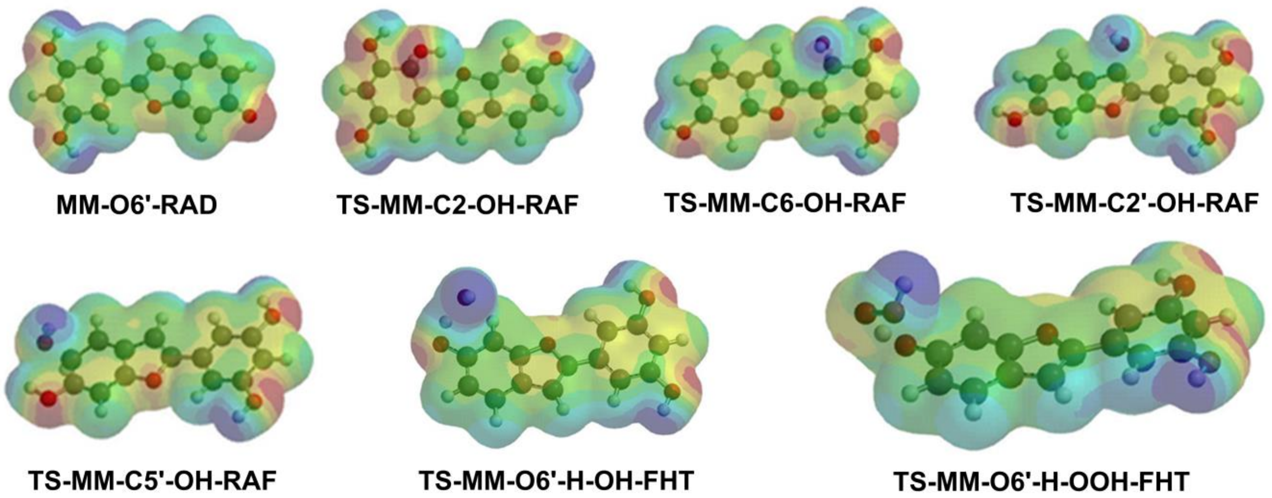

Figure S1: The density surface of the typical TSs and radicals according to FHT and RAF

References

1. M. G. Evans and M. Polanyi, *Trans. Faraday Soc.*, 1935, **31**, 875-894.
2. H. Eyring, *J. Chem. Phys.*, 1935, **3**, 107-115.
3. D. G. Truhlar, W. L. Hase and J. T. Hynes, *J. Phys. Chem.*, 1983, **87**, 2664-2682.
4. T. Furuncuoglu, I. Ugur, I. Degirmenci and V. Aviyente, *Macromolecules*, 2010, **43**, 1823-1835.
5. E. Vélez, J. Quijano, R. Notario, E. Pabón, J. Murillo, J. Leal, E. Zapata and G. Alarcón, *J. Phys. Org. Chem.*, 2009, **22**, 971-977.
6. E. Pollak and P. Pechukas, *J. Am. Chem. Soc.*, 1978, **100**, 2984-2991.
7. A. Fernández-Ramos, B. A. Ellingson, R. Meana-Pañeda, J. M. Marques and D. G. Truhlar, *Theor. Chem. Acc.*, 2007, **118**, 813-826.
8. C. Eckart, *Phy. Rev.*, 1930, **35**, 1303.
9. R. A. Marcus, *Annu. Rev. Phys. Chem.*, 1964, **15**, 155-196.
10. R. A. Marcus, *Rev. Mod. Phys.*, 1993, **65**, 599.
11. Y. Lu, A. Wang, P. Shi and H. Zhang, *PloS one*, 2017, **12**, e0169773.
12. Y. Lu, A. Wang, P. Shi, H. Zhang and Z. Li, *PloS one*, 2015, **10**, e0133259.
13. S. F. Nelsen, S. C. Blackstock and Y. Kim, *J. Am. Chem. Soc.*, 1987, **109**, 677-682.
14. S. F. Nelsen, M. N. Weaver, Y. Luo, J. R. Pladziewicz, L. K. Ausman, T. L. Jentzsch and J. J. O'Konek, *J. Phys. Chem. A*, 2006, **110**, 11665-11676.
15. A. Galano and J. R. Alvarez-Idaboy, *J. Comput. Chem.*, 2013, **34**, 2430-2445.
16. F. C. Collins and G. E. Kimball, *J. Colloid Sci.* , 1949, **4**, 425-437.
17. M. Von Smoluchowski, *Z. Phys. Chem*, 1917, **92**, 129-168.
18. D. G. Truhlar, *J. Chem. Educ.*, 1985, **62**, 104.
19. A. Einstein, *Ann. Phys.*, 1905, **17**, 549-560.
20. G. G. Stokes, *Mathematical and Physical Papers*, University Press, Cambridge, 1905.
21. A. Galano and J. Raúl Alvarez-Idaboy, *Int. J. Quantum Chem.*, 2019, **119**, e25665.
22. Q. V. Vo, T. V. Gon, M. V. Bay and A. Mechler, *J. Phys. Chem. B*, 2019, **123**, 10672-10679.
23. Q. V. Vo and A. Mechler, *J. Chem. Inf. Model.*, 2020, **60**, 316-321.
24. Y. Okuno, *Chem.: Eur. J.*, 1997, **3**, 212-218.
25. S. Benson, *The foundations of chemical kinetics:* , Malabar, Florida, 1982.
26. C. Iuga, J. R. Alvarez-Idaboy and A. Vivier-Bunge, *J. Phys. Chem. B*, 2011, **115**, 12234-12246.
27. J. R. Alvarez-Idaboy, L. Reyes and N. Mora-Diez, *Org. Biomol. Chem.*, 2007, **5**, 3682-3689.
28. T. H. Le, T. T. Tran and L. K. Huynh, *Chemom. Intell. Lab. Syst.*, 2018, **172**, 10-16.
